# Reply: Moving Beyond the Perfect Donor: An Interdisciplinary Approach to Optimizing Donor Utilization

**DOI:** 10.1016/j.jacadv.2025.102321

**Published:** 2025-11-11

**Authors:** Aditi Venkatraman, Yasbanoo Moayedi

**Affiliations:** a Ajmera Transplant Centre, University Health Network, Toronto, Ontario, Canada; b University of Toronto, Toronto, Ontario, Canada

We thank Kakoudaki et al for their thoughtful comments on our recent study.^1^ We appreciate their interest and commend the excellent outcomes achieved at Royal Papworth Hospital.

Their experience mirrors many of the approaches to donor utilization we have implemented at the Ajmera Transplant Centre. Both centers employ shared decision-making between at least 2 clinicians, structured group reviews to promote consistency and accountability. Their comprehensive weekly review process which integrates both center- and nation-level data is similar to our core group review is an excellent example of how multidisciplinary peer review can enhance practice and foster culture of continuous learning.

We agree that careful data review, reflective practice, and collaborative scrutiny are essential to donor utilization. It is encouraging to see parallel processes are achieving improvements in utilization without compromising safety across different health systems. Since this publication, we have had programs reach out requesting dashboard templates to help standardize this dialogue. We would be pleased to share our code with interested centers and invite colleagues to contact us directly should they wish to implement similar tools.

We look forward to continued global dialogue, where scalable, data-driven, multidisciplinary strategies can strengthen donor decision-making and optimize outcomes for transplant programs worldwide.
